# The Role of an Interventional Program for Improving Pharmacovigilance at a Pediatric Facility

**DOI:** 10.3389/fphar.2019.01004

**Published:** 2019-09-10

**Authors:** Nirit Kronenfeld, Shirly Gamsu, Rimona Keidar, Ayelet Livneh, Matitiahu Berkovitch, Michael Goldman, Hilla Bahat, Miriam Shchory-Potlog, Tomer Ziv-Baran, Ibrahim Abu-Kishk

**Affiliations:** ^1^Pharmacy Department, Shamir Medical Center (Assaf Harofeh), Zerifin, Israel, affiliated to the Sackler Faculty of Medicine, Tel-Aviv University, Tel-Aviv, Israel; ^2^Pediatric Department, Shamir Medical Center (Assaf Harofeh), Zerifin, Israel, affiliated to the Sackler Faculty of Medicine, Tel Aviv University, Tel Aviv, Israel; ^3^Neonatal Intensive Care Unit, Shamir Medical Center (Assaf Harofeh), Zerifin, Israel, affiliated to the Sackler Faculty of Medicine, Tel-Aviv University, Tel-Aviv, Israel; ^4^Clinical Pharmacology and Toxicology Unit, Shamir Medical Center (Assaf Harofeh), Zerifin, Israel, affiliated to the Sackler Faculty of Medicine, Tel-Aviv University, Tel-Aviv, Israel; ^5^Shamir (Assaf Harofeh) Academic School of Nursing, Zerifin, Israel, affiliated to the Henrietta Szold Hadassah-Hebrew University School of Nursing, Jerusalem, Israel; ^6^Department of Epidemiology and Preventive Medicine, School of Public Health, Sackler Faculty of Medicine, Tel-Aviv University, Tel-Aviv, Israel; ^7^Pediatric Intensive Care Unit, Shamir Medical Center (Assaf Harofeh), Zerifin, Israel, affiliated to the Sackler Faculty of Medicine, Tel-Aviv University, Tel-Aviv, Israel

**Keywords:** adverse drug reactions, pharmacovigilance, pediatric, intervention, sides effects, reporting

## Abstract

**Background:** The reporting rate of adverse drug reactions (ADRs) by healthcare professionals is low. ADR interventional programs may improve the reporting rate by the medical team. Our literature search revealed that only a few interventional studies among the pediatric population have been published.

**Objective:** We aimed to create an interventional program in order to improve the reporting rate of ADRs during the interventional period compared to the control period, detect which drugs frequently lead to ADRs and determine the most serious ADRs.

**Design:** A 3-month prospective intervention study compared with one year prior to the intervention (control period). ADR data was also collected for the year following the study period. Healthcare professionals were encouraged to report ADRs and an ADR reporting system was created for them.

**Setting:** Pediatric Division at Shamir Medical Center (Assaf Harofeh), a tertiary care medical center.

**Results:** The study population included 3,753 admitted patients with 1,323 prescriptions during the study period. During the period before the intervention was started, the ADR reporting rate was null. During the study period, 46 reports were collected: 46% from the general pediatric department, 26% from the pediatric neurology department, and 22% and 6% from the pediatric and neonatal intensive care units, respectively. Antiepileptic medications, IVIG, steroids and antibiotics were frequently reported to induce ADRs. Serious ADRs were also reported in 5 cases. One year of follow up after the intervention revealed a significant decline in the reporting rate.

**Conclusion:** It is important to periodically encourage healthcare professionals to report any ADRs in order to increase knowledge about medication safety and prevent fatal harm.

## Introduction

ADRs have a considerable adverse impact on the health of the population since a significant proportion of them are life-threatening or fatal. In a meta-analysis of 39 prospective studies from US hospitals, it has been shown that ADRs rank from the fourth to sixth leading cause of death (Lazarou et al., 1998). ADRs also have consequences in terms of costs, especially in hospitals (Edwards and Aronson, 2000; Gautier et al., 2003; Pirmohamed et al., 2004). Numerous factors influence ADR susceptibility, including multiple drug therapy, disease severity, age, and the type and number of drugs prescribed.

A new medicine must pass three hurdles before its approval by the national drug regulatory authority. Sufficient evidence is required to show the new drug to be of good quality, effective, and safe. Whereas the first two criteria must be met before considering approval, the issue of safety is less certain. Safety is not absolute, and it can be judged only in relation to efficacy, requiring judgment on the part of the regulators in deciding on acceptable safety limits. There is the possibility that rare yet serious adverse events will not be detected during the pre-registration development of the drug because of multiple reasons such as younger patients in comparison to the post marketing stage, a limited number of medications that are taken for a short period of time and a small sample size which is insufficient for presenting results with significant statistics.

National and international pharmacovigilance programs, intended to detect post marketing adverse drug reactions (ADRs), are based on spontaneous reports of ADRs by medical professionals. While spontaneous reporting remains a cornerstone of pharmacovigilance in the regulatory environment, and is vital for detection, more systematic and robust epidemiological methods that take into account the limitations of spontaneous reporting are required.

In hospitals, reports of ADRs have become an important component of monitoring and evaluating activities performed. This information may be useful for identifying and minimizing preventable ADRs, while generally enhancing the ability of prescribers to manage ADRs more effectively (Wu and Pantaleo, 2003).

In Israeli hospitals, as in many other countries, reporting of severe or life threatening ADRs by doctors is compulsory. Doctors are required to report ADRs to the Ministry of Health, and ADRs in hospitals must be reported to the hospital pharmacy. In Israel, reports to the Ministry of Health can be sent by mail, or by filing a web based form on an ADR reporting site manned by the Israeli Society of Clinical Pharmacology, to which the Ministry of Health has access. Well established sites like the FDA Medwatch are lacking in Israel (Goldstein et al., 2013). ADR interventional programs may improve the reporting rate by the medical team. Our literature search revealed that only a few interventional studies among the pediatric population have been published.

## Materials and Methods

An adverse drug reaction (ADR) is defined by the World Health Organization (WHO) as any noxious, unintended, or undesired effect of a drug that occurs at doses used in humans for prophylaxis, diagnosis, or therapy (World Health Organization, 2002). A serious adverse event or reaction is any untoward medical occurrence in a patient associated with the use of a drug that results in death, is life threatening, requires inpatient hospitalization or prolongation of existing hospitalization, or results in persistent or significant disability or incapacity (World Health Organization, 2002).

The research was approved by the Helsinki Committee of Shamir Medical Center (Assaf Harofeh) and took place in the Pediatric Division. Shamir Medical Center (Assaf Harofeh) is an 850-bed teaching hospital in central Israel, treating an urban and rural population of approximately 1 million people. The Pediatric Division is a comprehensive 100-bed childcare facility comprising general pediatric units, pediatric sub-specialties, pediatric general and orthopedic surgery units, a daycare clinic and a general pediatric intensive care unit. In addition, there is a 72-bed regular care new born nursery, a 30-bed newborn special care department (which includes a 10-bed neonatal intensive care unit), and a unique pediatric neurology and rehabilitation referral center. The average annual number of children hospitalized in the Pediatric Division is approximately 15,000 (∼2,500 monthly). The staff involved in the study included 33 physicians and 98 nurses. The intervention was made in the hospital so the patients that included were only hospitalized. The monthly number of prescriptions in the Pediatric Division is around 450.

A prospective study was conducted for 3 months (the interventional period). The interventional program included: placing posters in medical team rooms and nurse stations; supplying nurses with forms requiring them to fax reports of ADRs including minimum information (patient’s name, ID number and a short description of the suspected ADR); presenting a 45-minute lecture about the importance of pharmacovigilance to doctors and nurses in the Pediatric Division; distributing papers summarizing the main topics of the lecture to the medical team and nurses and; inserting a reporting paper into the patient’s medical record (**Table 1**).

**Table 1:** Presentation of the interventions list.

Ordinal Number	Type of intervention
1	Placing posters in medical team rooms and nurses’ stations.
2	Supplying nurses with forms requiring them to fax reports of ADRs.
3	Presenting a 45-minute lecture about the importance of pharmacovigilance to doctors and nurses.
4	Distributing papers summarizing the main topics of the lecture to the medical team and nurses
5	Inserting a reporting paper into the patient’s medical record.

The first part of the presentation included definitions of pharmacovigilance and ADRs; a review of international studies on drug-related morbidity and mortality, hospital admissions, and cost to health systems and patients; and a description of the methods used in pharmacovigilance and in spontaneous reporting systems, explaining that underreporting constitutes the system’s principal limitation. The second part addressed the attitudes associated with underreporting, emphasizing that only 5 min are required to complete the report form. In addition, a reminder card similar to the report form and containing the principal messages of the presentation was distributed to approximately 90% of the physicians attending the sessions.

The medical staff was encouraged to report anything they suspected, even if the ADR was common or mild in nature or uncertain. During the study period, notifications were sent *via* a text message every week. Emails were sent to the network members (every nurse and physician in the pediatric division who has an outlook email), amusingly and pleasantly reminding them to report ADRs. The letters were written attractively using colors, different fonts, rhymes, idioms, etc. They also contained information on one or two of the latest ADRs reported, emphasizing the lesson learned from each report. The emails were sent to a group established on the Outlook Mailing Software, listing all the members of the network, at no particular time of day, and on no particular day of the month. Clinical meetings with hospital healthcare professionals raised awareness of ADR monitoring and its importance.

The details required for reporting were the patient’s name, ID number and a short description of the ADR.

The reports were sent to the Unit of Pediatric Pharmacology, as was customary before the study period. A trained pharmacist was in charge of documenting all the ADRs on the patient’s chart and the pharmacy plan of action. The Pediatric Pharmacology Unit subsequently sends the reports to the Israeli Ministry of Health. The ADRs reported during the study period (February to April 2016) were compared to the ADRs reported during the year prior to and after the study period. The rates of reporter role (doctors vs. nurses), type of ADR (allergic reaction vs. side effect), severity of ADR (mild vs. moderate vs. severe) and ADR in accordance with specific medication classes were examined.

## Results

The study was conducted in a number of departments in the Pediatric Division: General Pediatric Department, Pediatric Neurology Department, Pediatric Intensive Care Unit (ICU) and Neonatal Intensive Care Unit (NICU). The study population included 3,753 admitted patients with 1,323 prescriptions during the intervention period. There was no significant difference of the occupancy and the number of prescriptions in the pediatric departments before, during and after the intervention period.

During the year before the intervention period no ADR was reported. In the study period, the rate of reporting ADRs rose significantly to 46 reports (mean of 15.3 reports per month). During 6 months after the study period, the ADR reporting rate was 20 (mean of 3.3 reports per month). In the period 6–12 months after the intervention period, no ADR reports were received (**Table 2**). Sixty-five percent of the ADRs were reported by physicians and 35% were reported by nurses.

**Table 2:** Number of ADRs reported before, during and after the study period.

Department	ADR reports*				
	Prior to intervention	During intervention n (%)	<6 months after intervention n (%)	6–12 months after intervention	Total ADRs reported n (%)
General pediatric	0	21 (46)	11 (55)	0	32 (48.5)
Pediatric neurology	0	12 (26)	8 (40)	0	20 (30.3)
PICU	0	10 (22)	0	0	10 (15.2)
NICU	0	3 (6)	1 (5)	0	4 (6)
Total ADRs reported	0	46	20	0	66

ADR, adverse drug reaction; PICU, pediatric intensive care unit; NICU, neonatal intensive care unit.

*Each report represents a single ADR.

**Figure 1:**
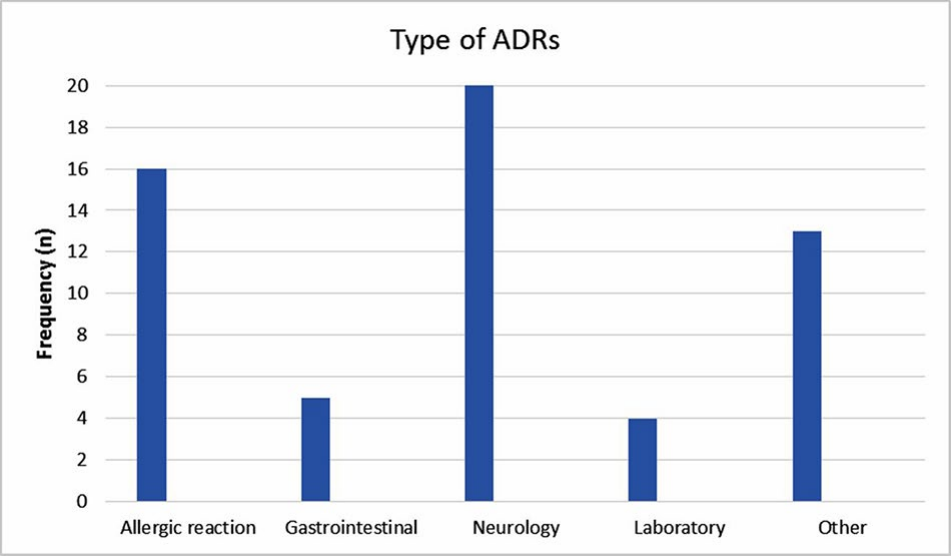
Frequency of adverse drug reactions.

The reported ADRs were classified as neurologic, gastrointestinal, allergic, laboratory and other reactions. The most reported ADRs were allergic reactions and neurologic adverse drug reactions (**Figure 1**). The medication groups involved in the ADRs are summarized in **Table 3**.

**Table 3:** Number of ADRs reported according to therapeutic category.

Therapeutic category	ATC code	Number of reported ADRs (%)
Antiepileptic drugs	N03	20 (30)
Antibiotics	J01	14 (21)
Sedatives	N01	9 (14)
Steroids	H02	4 (6)
Intravenous immunoglobulins	L03	2 (3)
Other		17 (26)

ADR, adverse drug reaction; ATC, anatomical therapeutic chemical classification.

Reports of serious adverse reactions included two cases of apnea when using intravenous immunoglobulins (IVIG). In one case IVIG was administered as a treatment for immune neonatal jaundice that developed during the first hour of life. The IVIG was received *via* a slow intravenous infusion, 30 min later apnea and desaturation were observed, the IVIG infusion was stopped and the infant was transferred to the NICU for monitoring. During his stay at the NICU no clinical events were observed. A similar scenario was also observed in the second case, which was treated with IVIG for early jaundice. The neonate developed cyanosis and apnea that required mechanical ventilation, the IVIG infusion was stopped. In addition, 3 cases of ataxia, shivering and dizziness were reported after using Clobazam (Frisium^®^) at doses recommended in the literature. In 2 cases, the medication was stopped and in the third case the dose was reduced and the ADRs passed immediately after.

## Discussion

This study showed a dramatic increase in reporting ADRs during the intervention period, a trend that decreased gradually and returned to the baseline after 1 year of follow up. The most reported ADRs were allergic reactions and neurologic adverse drug reactions; 7.5% of the ADRs were severe. The most common drugs involved were antiepileptic, antibiotic and sedative drugs.

During the intervention period, doctors became aware more than before about the importance of preventing errors in prescribing medications. During the medical meeting, medical prescribing errors were presented, and the doctors discussed actions needed to prevent these errors. Nurses began to examine the medical prescriptions and detected errors, most of the time, before the medications were given to the patients. Interestingly, the reports decreased significantly after the intervention period, suggesting the need for persistent acts to increase the awareness of the medical and paramedical team in terms of ADRs.

Figueiras et al. (2006) conducted a cluster-randomized controlled trial that developed a continuing medical education multifaceted intervention, comprising an outreach visit, reminder card, and report form. The outreach visit consisted of a 1-hour 2-part presentation that took place during weekly staff meetings to ensure that the greatest number of physicians could be present. The intervention visits were conducted from March 2004 through July 2004. Compared with the control group, at baseline, the intervention group had lower rates of reporting for all categories of ADR, measured by reports per 1,000 physician-years; however, none of these was statistically significant. Comparing total ADR reporting, the intervention group increased from 7.6 (95% CI, 4.0–12.6) at baseline to 100.2 (95% CI, 85.2–116.4) in the post intervention period, while the control group increased from 11.3 (95% CI, 8.9–14.1) to 14.5 (95% CI, 12.0–18.0), respectively (P < 0.001). The reporting rate in the intervention group increased sharply in the initial phase of the intervention, and the RR for total ADR reporting in the first 4-month period after the intervention was 27.78 (95% CI, 8.36–92.23; P < 0.001). The magnitude of the effect decreased in subsequent periods, remaining statistically significant throughout the first 12 months after the intervention.

In another interventional study conducted by McGettigan et al. (1997), the interventional program was to make yellow reporting cards prominently available and place one in each patient’s chart upon admission. In addition, doctors were regularly reminded that ADRs should be reported. Over 3 months of intervention, the greater availability of yellow cards and reminders about reporting ADRs led to an approximate five-fold increase in reports, but reporting declined rapidly thereafter when verbal reminders were withdrawn, despite continued ready availability of cards suggesting that making cards available alone does not significantly increase reporting. In our study, a number of types of intervention were made, in addition to repeated verbal and electronic notifications. However, it was difficult to determine which type of intervention was effective. We suggest periodically implementing all the interventions used in the current study in order to maintain a high rate of ADR reports, such as posters in medical team rooms, nurses’ stations, requirement to fax reports of ADRs, periodic lectures about the importance of pharmacovigilance to doctors and nurses, insertion of papers that summarize the main topics of the lectures and a reporting paper into the patients’ medical records.

Recently, a meta‐analysis tried to find factors responsible for underreporting and found; ignorance (only severe ADRs need to be reported), diffidence (fear of appearing ridiculous for reporting merely suspected ADRs), lethargy (lack of time or other excuses), indifference (the one case could not contribute to medical knowledge), insecurity (it is nearly impossible to determine whether a drug is responsible for an adverse reaction), and complacency (only safe drugs are allowed on the market (Lopez-Gonzalez et al., 2009).

When a new drug is licensed, drug safety information tends to be limited. Before approval, drugs are usually evaluated for a defined indication in clinical trials of relatively short duration and involving a small sample size. Study populations often exclude patients with complicated medical conditions, those receiving concurrent drug therapy, and young and elderly persons (Ioannidis and Lau, 2001; Psaty et al., 2004). Hence, after a drug is marketed, previously unidentified important adverse drug reactions (ADRs) may occur. Post marketing surveillance is important for the discovery of such new ADRs, and a spontaneous reporting system is the primary method of post marketing surveillance (Ahmad, 2003; Strom, 2004; Wysowski and Swartz, 2005; Lexchin, 2006).

Physicians are in a position to play a key role in reporting programs (Ahmad, 2003; Wysowski and Swartz, 2005), but underreporting is very common, with an estimated median underreporting rate of 94% (Hazell and Shakir, 2006). This can delay detection of important ADRs. Studies from different settings indicate inadequate knowledge about ADRs among physicians, as well as attitudes that are associated with a high degree of underreporting (Williams and Feely, 1999; Perlík et al., 2002).

Regarding information about adverse drug reactions reported in children, Aagaard et al. (2010a) identified a few studies monitoring ADRs in general pediatric populations and found a higher prevalence rate of ADR reports among hospitalized children and outpatients than in national databases. They suggested that there is a considerable potential for raising the rate of ADR reports by advocating spontaneous reporting systems. In another study by Aagaard et al. (2010b) reports of ADRs in the pediatric population were examined in Denmark and they found that there was a decline of ADR reports during a decade. These two works illustrate several points that are consistent with the findings of the current study; the ADR reports are inconsistent and can be improved by the presence of supporting systems.

Most of the reported ADRs reported in the current study were due to antiepileptic drugs. In some of the cases, patients required hospitalization, in other cases the antiepileptic medication which caused an ADR was substituted by another. Other drugs were also involved and caused several reactions. However, 7.5% of the ADRs were severe; in one case mechanical ventilation was required due to IVIG administration, and this rare ADR was previously reported (Kumar et al., 2014).

A literature review of ADRs in pediatric patients found that antibiotics are involved in the majority of the cases (up to 83%), followed by nervous system medications and respiratory system medications (up to 35% each) (Bourgeois et al., 2009; Aagaard et al., 2010a). In the current study, antibiotics came second (21%) after nervous system medications. This can be partially explained by the uniqueness of our Pediatric Neurology Unit. This unit is a referral center for refractory epilepsy and the majority of the admitted patients are treated with multidrugs.

This study has limitations due to the short period of intervention and the lack of assessment of unreported ADRs during the intervention period. Information about causality is also lacking (it is a descriptive study) and a small number of reports were obtained. However, this study is one of the few studies that have been designed as interventional on a pediatric population. An additional strength of this study is that a structured intervention was established in the hospital and a follow-up period was implemented.

## Conclusion

In this study, an increased reporting rate of ADRs was observed during the interventional period. The reports then decreased gradually after this period when the interventions were ceased. Twelve months from the study period, no spontaneous ADR reports were received by the medical team. These results emphasize the importance of conducting interventions regularly in order to encourage medical staff to report ADRs spontaneously.

## Data Availability

The datasets generated for this study are available on request to the corresponding author.

## Ethics Statement

This study was approved by the ethical committee of Shamir Medical Center (Assaf Harofeh).

## Author Contributions

MB, MS-P, and IA-K contributed to conception and design of the study. NK and SG contributed to data collection. RK, AL, MG, HB and TZ-B contributed to data analysis and interpretation. NK, SG, RK, AL, MG, HB, MS-P and TZ-B contributed to drafting the paper. MB and IA-K contributed to critical revision of the paper for important intellectual content. All authors read and approved the final version of the manuscript.

## Conflict of Interest Statement

The authors declare that the research was conducted in the absence of any commercial or financial relationships that could be construed as a potential conflict of interest.
